# BORDERLINE LEPROMATOUS LEPROSY WITH NEUROFIBROMATOSIS

**DOI:** 10.4103/0019-5154.70678

**Published:** 2010

**Authors:** Gnaneshwar Rao Angoori, Indira Danturty, T N Rekha Singh

**Affiliations:** *From the Department of Dermatology, Gandhi Medical College, Secunderabad, India*

**Keywords:** *Leprosy*, *neurofibromata*, *café-au-lait macules*

## Abstract

The coexistence of leprosy with neurofibromatosis is rare both the diseases present with nerve thickening and skin lesions (patches and nodules). The coexistence of neurofibroma with borderline tuberculoid, lepromatous, histoid, and neuritic leprosy has been reported in the past. We report here a case of borderline lepromatous leprosy coexisting with neurofibromatosis in a 60 year-old male, who presented with neurofibromata and nerve thickening. Histopathology of skin biopsy from the leprosy and neurofibroma nodules confirmed the diagnosis of leprosy and neurofibroma.

## Introduction

Involvement of nerve and skin occurs in leprosy and neurofibromatosis, with Schwann cells being the primary target for both diseases. However, the etiology and pathophysiology of both these diseases are different with leprosy being an infection with *Mycobacterium leprae* and neurofibromatosis being a genodermatosis. We report here a case of neurofibromatosis associated with borderline lepromatous leprosy.

## Case Report

A 60 year-old male presented to the Skin Department of Gandhi Hospital with multiple swellings all over the body since his childhood. He gave a history of epistaxis and joint pains for the last one year. The nodules were small in size and gradually grew to attain the present size and involved the entire body. Past history was not contributory and family history was negative for neurofibromatosis and leprosy.

**Figure 1:**
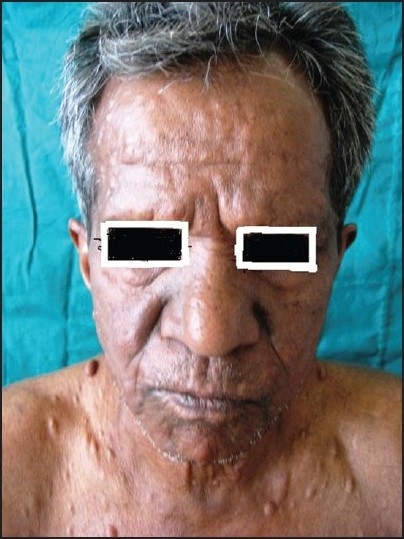
Infiltration of fore head and upper lip along with neurofibroma on the chest

**Figure 2:**
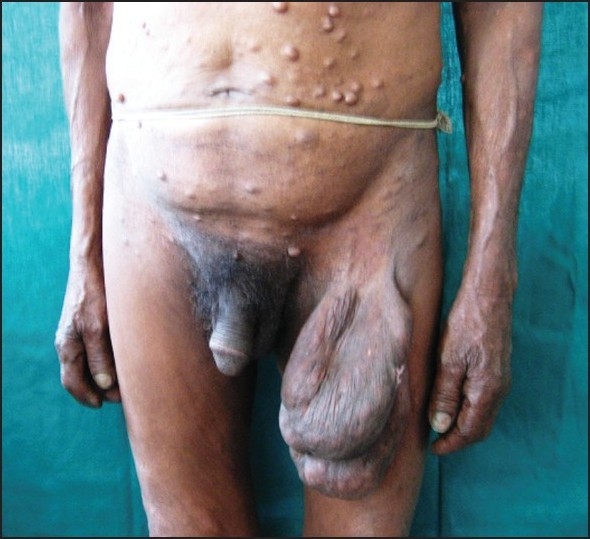
Large plexiform neurofibroma hanging from left thigh and multiple neurofibromas involving trunk along with multiple hypopigmented patches on the right thigh

Cutaneous examination revealed multiple, small, 1–2 cm, soft, nontender, dome-shaped nodules over the front of the chest, forehead, and back; infiltration on the forehead, and superciliary madarosis. [Figure 1] Both the feet were dry and fissured and five café-au-lait macules oval in shape and 3–7 cm in size were present on the back, left lower part of the abdomen, left inguinal region, and the upper part of the left thigh. Multiple hypopigmented macules with sensory deficit were present over the back of the trunk and front of the thighs. A soft, nontender, diffuse, pendulous swelling 15–20 cm in size hanging from the medial aspect of the left thigh was also present [Figure 2]. Bilateral ulnar and radial cutaneous nerves and left lateral popliteal and left sural nerves were thickened and nontender. Motor system was normal but an examination of the cardiovascular system revealed an ejection systolic murmur radiating to the axilla that was associated with thrill. He was clinically diagnosed to have borderline lepromatous leprosy with neurofibromatosis.

**Figure 3:**
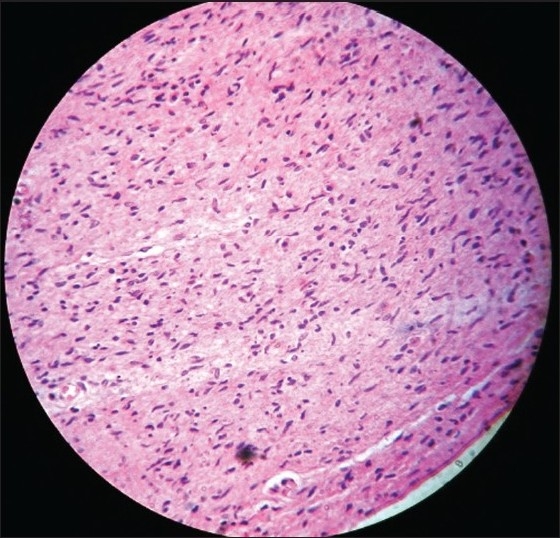
Histopathology of neurofibroma nodule showing spindlecells with eosinophilic cytoplasm and wavy nuciei (H and E stain, ×400)

**Figure 4:**
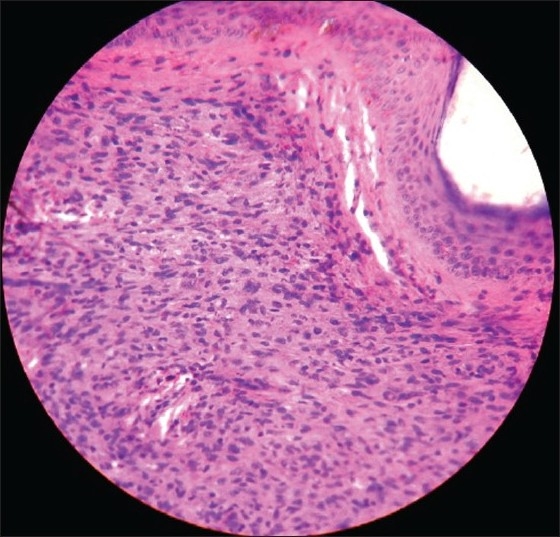
Histopathology of Skin shows Grenz zone and diffuse macrophage granuloma (H and E Stain, ×400)

**Figure 5:**
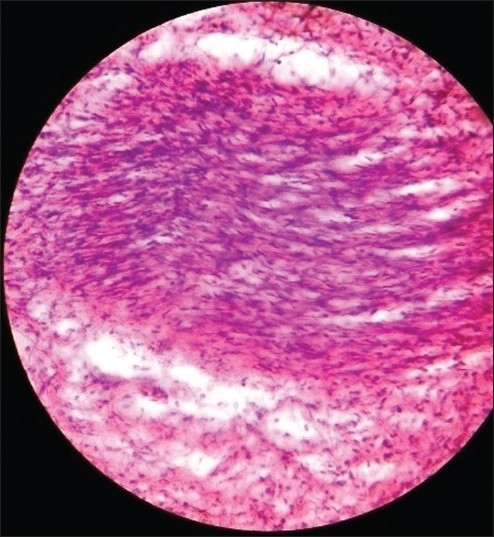
Histopathology of plexiform neurofibroma showing thickened nerves embedded in the back ground of neurofibromatous matrix (H and E stain, ×400)

A slit skin smear from the ear lobule revealed a bacillary load of 3+. Biopsy taken from the neurofibroma nodule showed spindle cells with eosinophilic cytoplasm and wavy nuclei [Figure 3] and was immunohistochemically positive for S-100. Biopsy from the plexiform neurofibroma showed thickened nerves embedded in the background of a neurofibromatous matrix. [Figure 4] Biopsy from the leprosy nodule showed Grenz zone and diffuse macrophage granoloma. [Figure 5] Fite faraco stain showed a few acid-fast bacilli in the granuloma in the dermis.

## Discussion

Neurofibromatosis and leprosy affect Schwann cells and electron microscopic studies have demonstrated that most cells in neurofibromatosis are derived from Schwann cells. It has also been shown that it is the Schwann cell and not the axon that is predominantly (or possibly even solely) invaded by M. *leprae*.[1]

The coexistence of leprosy and neurofibromatosis in the same patient is a rare and an interesting finding and can pose a diagnostic dilemma. Both conditions may present with nodules and nerve thickening[2,3] and with leprosy being mistaken for neurofibromatosis in non endemic areas, appropriate treatment has been delayed. Conversely, neurofibromatosis has been mistaken for leprosy in a population prone to leprosy.[4] Mittal *et al*. reported a case of neurofibromatosis with gross enlargement of all peripheral nerve trunks simulating leprosy.[5] Khandpur *et al*. reported an unusual hypopigmentation strikingly limited to the neurofibromas with symmetrical nerve enlargement, evoking a strong clinical suspicion of coexistent lepromatous leprosy. However, leprosy was ruled out by microbiological, histopathological, and electrophysiological studies.[6] Swift has reported two cases of neurofibromatosis with lepromatous leprosy.[1] He found preferential localization of lepra bacilli within the tumor cells (neurofibroma). Neurofibromatosis has earlier been reported to coexist with lepromatous,[7] histoid,[8] pure neuritic,[9] and borderline tuberculoid leprosy.[10]
